# Strategies for Reliable Stress Recognition: A Machine Learning Approach Using Heart Rate Variability Features

**DOI:** 10.3390/s24103210

**Published:** 2024-05-18

**Authors:** Mariam Bahameish, Tony Stockman, Jesús Requena Carrión

**Affiliations:** 1 College of Science and Engineering, Hamad Bin Khalifa University, Doha P.O. Box 34110, Qatar; 2 School of Electronic Engineering and Computer Science, Queen Mary University of London, London E1 4NS, UK; t.stockman@qmul.ac.uk (T.S.); j.requena@qmul.ac.uk (J.R.C.)

**Keywords:** heart rate variability, stress recognition, affective computing, machine learning

## Abstract

Stress recognition, particularly using machine learning (ML) with physiological data such as heart rate variability (HRV), holds promise for mental health interventions. However, limited datasets in affective computing and healthcare research can lead to inaccurate conclusions regarding the ML model performance. This study employed supervised learning algorithms to classify stress and relaxation states using HRV measures. To account for limitations associated with small datasets, robust strategies were implemented based on methodological recommendations for ML with a limited dataset, including data segmentation, feature selection, and model evaluation. Our findings highlight that the random forest model achieved the best performance in distinguishing stress from non-stress states. Notably, it showed higher performance in identifying stress from relaxation (F1-score: 86.3%) compared to neutral states (F1-score: 65.8%). Additionally, the model demonstrated generalizability when tested on independent secondary datasets, showcasing its ability to distinguish between stress and relaxation states. While our performance metrics might be lower than some previous studies, this likely reflects our focus on robust methodologies to enhance the generalizability and interpretability of ML models, which are crucial for real-world applications with limited datasets.

## 1. Introduction

Affect recognition constitutes a critical element in discerning internal bodily feelings (e.g., fear, happiness, and stress) that influence mental health and well-being [1]. Traditionally, mental health has been evaluated using standardized self-report instruments with established clinical validity, such as the Patient Health Questionnaire (PHQ-9) for depression assessment [2]. However, these questionnaires are susceptible to subjective bias, as respondents may provide inaccurate or imprecise answers [3]. Fortunately, questionnaires can be supported by physiological data to provide a reliable approach for determining an individual’s mental state. The concept of inferring mental states from physiological data is not new, dating back to the 1920s with the invention of the lie detector, which functioned by sensing changes in blood pressure, breathing, and heart rate [4]. In fact, advancements in wearable technology have facilitated the development of more advanced affect recognition and health monitoring systems. This allows for the continuous monitoring of physiological data, offering the potential to identify early warning signs for mental disorders [5].

Given the complexity of psychophysiological responses, myriad studies have examined the development of affect detection and recognition prototypes using machine learning (ML). These techniques encompass supervised and unsupervised learning approaches. ML offers a powerful framework for solving classification and recognition problems, demonstrating remarkable success in diverse fields, particularly clinical applications [6,7]. Pioneering research by Picard et al. [1] shifted the focus from facial and verbal expressions to physiological responses for affect recognition. Using data from a single participant over several weeks, this study achieved a classification performance of 81% for eight emotions based on breathing, heart activity, muscle activity, and skin conductance. This pivotal work paved the way for subsequent studies employing ML algorithms with multiparticipant data to recognize various affective states, including emotions [8,9,10], fear [11,12], and stress [13,14].

Recognizing different stress levels holds significant promise for developing early intervention strategies, stress management techniques, and preventative measures to promote mental health and well-being [15]. A growing body of research explores stress detection through the development of predictive models using ML algorithms based on physiological data [13,14,15,16,17,18,19]. Among various physiological measures, heart rate variability (HRV) has emerged as a critical biomarker for monitoring stress responses. HRV reflects the activity of the autonomic nervous system, providing valuable insights into stress regulation [20,21,22].

Affective computing and healthcare research often rely on limited datasets, necessitating caution when developing ML algorithms to prevent biased conclusions about model performance. Schmidt et al. [23] reviewed affect recognition using ML and found that most studies (43 out of 46) used data from fewer than 40 participants, with only one exceeding 100. Furthermore, the reported accuracy rates varied widely (40% to 97%), raising concerns in areas like biomedical research [24] and psychiatric studies [25]. Significant variations in accuracy due to limited data could potentially indicate overestimated performance or methodological shortcomings. These shortcomings manifest as issues with data segmentation, inappropriate feature selection, and an inadequate validation strategy.

The present study employed supervised learning algorithms for stress and relaxation classification using HRV measures. We accounted for limitations associated with small datasets, a prevalent challenge when implementing and interpreting ML algorithms as documented in the literature. Accordingly, our study design incorporates best practices for reliable ML algorithms with limited datasets [24,25,26,27,28].

## 2. Background

### 2.1. Related Work

ML techniques for stress detection have garnered significant interest in affective computing and healthcare [13,18,29,30]. Recent advancements in technology, especially wearable devices, have facilitated the non-invasive collection of physiological data. In a comprehensive review of affect recognition, Schmidt et al. [23] examined the detection of several affective states, including emotion, excitement, frustration, happiness, relaxation, and stress. Most of the studies (34 out of 46) focused on identifying stress levels (16 studies) and emotional states (18 studies). The results highlight the use of various physiological signals in the reviewed studies: 40 used cardiac activity, 35 used skin conductivity, 15 used miscellaneous signals (e.g., accelerometer data, muscle activity, respiration, and temperature), and seven used brain activity.

Building upon the seminal work of Healey and Picard [13], which demonstrated the feasibility of real-world driver stress detection using physiological data, researchers have increasingly explored ML algorithms for this purpose. The publicly available dataset from this foundational study has been instrumental in advancing the field, providing a valuable resource for algorithm development and validation. In parallel, researchers have introduced new datasets focused on monitoring physiological responses during cognitive stress tasks [31,32,33,34], thereby enriching the ML applications for affect recognition. For instance, Dalmeida and Masala [18] leveraged features extracted from HRV within one of these public datasets to train and evaluate various supervised ML algorithms for stress detection. Notably, their work explored the generalizability of these models by testing them on new HRV data collected via wearable devices. Similarly, Benchekroun et al. [35] conducted a cross-dataset analysis to assess the generalizability of HRV-based stress detection models. However, these studies had limitations, such as the selection of features irrelevant to the context of the investigated problem and the use of overlapping window segmentation to increase the dataset size. Focusing on HRV analysis, three standardized analytical approaches have been articulated by the Task Force of the European Society of Cardiology and the North American Society of Pacing and Electrophysiology [21]: time domain, frequency domain, and non-linear methods as summarized in Table 1 [36].

### 2.2. Methodological Limitations

The recent surge in affect recognition research using physiological data and ML algorithms has highlighted several methodological challenges. These challenges encompass issues with data segmentation, feature engineering, and model evaluation. Inadequate attention to these aspects can lead to overfitting, overly optimistic performance estimates, and issues with generalizability, thereby hindering both the deployment and interpretation of the developed ML models [24,25,26,27,28]. Additionally, researchers emphasize the need for explainable ML methods, particularly in healthcare applications, to improve user understanding of the models’ predictions and decision-making processes [37,38,39,40].

#### 2.2.1. Data Segmentation

**Figure 1:**
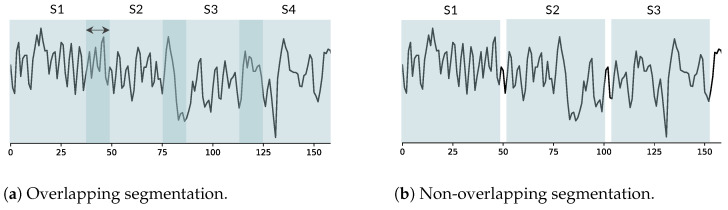
Physiological data segmentation approaches with a 50-second window size.

A critical issue arises when researchers seek to artificially increase dataset size by dividing each participant’s physiological data into multiple segments [18,41,42]. This practice violates the fundamental statistical assumption that observations must be independent since these resulting segments are interdependent due to being derived from the same participant. This can lead to data leakage, where dependent observations from the same participant are present in both the training and testing sets [24]. Furthermore, the use of overlapping window segmentation presents another potential source of dependency [31,43,44]. With this approach, observations not only come from the same participant but the physiological data themselves are partially shared across segments. Figure 1 illustrates an example of a 150 s HRV signal analyzed with a 50 s window size. This results in four segments with an overlapping approach (Figure 1a) and three segments with a non-overlapping approach (Figure 1b). For instance, a study investigating the detection of panic attack severity used overlapping windows on HRV data from 10 participants [45]. This approach generated a large number of observations (up to 1700 samples), substantially increasing the size of training and testing sets. A different study used overlapping windows with a 0.25 s shift on physiological data from 15 participants [31]. To address potential data leakage concerns arising from the segmentation process, they employed a subject-independent validation strategy.

In a fear classification study, Petrescu et al. [46] used overlapping and non-overlapping segmentation techniques on a dataset consisting of 32 participants. They reported equivocal results regarding the ML model performance for each segmentation approach. However, it is not clear to what extent the classification accuracy is impacted by the use of an overlapping technique vs. a non-overlapping one [47]. In fact, Dehghani et al. [48] demonstrated that improved model performance is associated with the use of dependent observations and the employment of an inadequate validation strategy. Data leakage can lead to overly optimistic estimates of a model’s generalizability because dependent observations are presented in both training and testing sets (refer to theoretical and mathematical derivations of performance overestimation [49,50]). One study addressed data leakage in mental stress classification by employing two key strategies to ensure data independence [17]. First, they avoided the use of any segmentation methods on the physiological data. Second, the study implemented a subject-independent validation strategy. This involved training and testing the ML models on separate groups of participants drawn from the same experiment. However, the generalizability of these findings remains limited due to the relatively small sample size.

#### 2.2.2. Feature Engineering

An additional issue relates to the number and choice of features employed in the ML classifiers. Inappropriate feature selection can lead to overfitting, where the model performs well on the training data but fails to generalize to unseen data. The existing literature highlights two suboptimal approaches to feature selection: (1) including all collected physiological measures, regardless of their relevance or dataset size, or (2) focusing solely on a limited set of features, potentially excluding relevant ones within the specific context of the investigated problem (e.g., behavioral and clinical; [31,51,52]).

Feature selection is a critical step in building robust ML models for healthcare applications. Including all collected physiological measures, regardless of relevance, can increase the dimensionality of the input space, thus increasing model complexity. This, as highlighted by Vabalas et al. [27], can lead to overfitting, especially in small datasets. Overfitting occurs when models memorize training data rather than learning generalizable patterns, resulting in poor performance on unseen data despite high training accuracy [53]. Consequently, a large number of features, especially redundant ones, can increase model complexity and hinder accurate ML performance evaluation [54]. Conversely, relying solely on statistical correlations for feature selection or mathematical-based algorithms for feature elimination does not provide a clear physiological rationale. While features with strong statistical associations might be identified, their clinical relevance remains questionable if they lack a sound physiological foundation. This can hinder model interpretability, making it difficult to understand the predictive mechanisms. In one example, non-linear HRV measures were selected to classify stress levels based on a statistical correlation analysis between the features and the target, but the physiological rationale behind the feature selection was not discussed [55]. Additionally, in another study, an analysis of 30 s segments was performed to obtain VLF power from the HRV frequency domain as an ML feature [18]. However, a segment with a minimum length of 5 min was found to be necessary for the robust computation of frequency components in the VLF band [36].

#### 2.2.3. Model Selection and Evaluation

A robust evaluation strategy is important in ensuring the generalizability of ML models, especially when dealing with small sample sizes. Several validation strategies are commonly used in the implementation of supervised ML algorithms, such as the hold-out method and cross-validation (CV) techniques [49]. The latter is more often employed in the context of limited datasets because of its ability to utilize the entire dataset in model fitting and evaluation.

K-fold is a prominent CV technique that randomly splits the dataset into K subsets and then trains the model iteratively on the K-1 subsets while keeping the remaining subset for validation [49]. Subsequently, overall performance is calculated as the average accuracy rate resulting from all K trials. However, random splitting with dependent observations poses a data leakage problem, as the training and validation sets may include data segments from the same participant. As briefly discussed in the previous sections, data leakage leads to biased and overly optimistic generalization performance estimates. Recent research has suggested splitting the data per participant using a subject-independent CV, such as the leave-one-out CV, to limit the effect of the dependent observations on the development and evaluation of the ML models [48,56]. The leave-one-out CV is an example of the K-fold method, where K is the total number of observations or participants. In a review of affect recognition, 13 studies (out of 46) used the K-fold CV, while the remaining studies incorporated variations of the leave-one-out CV [23]. This indicates that the leave-one-out CV is the preferred approach to mitigate the violation of the independence assumption within the context of affective computing applications. However, there are two key limitations of leave-one-out CV compared to k-fold CV. First, leave-one-out CV can be computationally expensive for large datasets, as it requires training the model n times (where n is the number of observations). Second, it is prone to high variance in performance estimates, particularly when outliers are present in the dataset.

Hyperparameter selection is commonly performed prior to model evaluation, although the use of a standard CV procedure with both processes can cause model selection bias. In particular, the use of the same validation set in each process can introduce overly optimistic estimates of the expected generalization performance [50]. Consequently, the nested CV technique can be used to manage both model evaluation and hyperparameter selection as integral processes, albeit with different validation sets.

### 2.3. Recommendations

This section provides practical recommendations to mitigate the risks associated with data leakage, overfitting, and performance overestimation in small datasets [24,25,26,27]:

Feature selection —Features should be rationally selected based on the clinical or physiological motivation of the investigated ML problem to facilitate the contextual interpretation of the model’s performance [57]. After determining the most relevant features, several techniques can be used for feature selection, such as correlational analysis or feature elimination methods. To minimize the effect of performance overestimation and reduce computational costs, the selected features should be limited to a reasonable feature-to-sample ratio [27]. A common practice in biomedical research using small datasets is to choose one feature for every 10 independent observations [24].
Validation strategy —Independence among observations should be considered when dealing with data generated from the same participant or obtained from data segmentation to avoid data leakage during model selection, particularly when splitting the dataset into training and validation/testing sets. Hence, an appropriate validation strategy should be implemented. The leave-one-out CV technique is notably effective for small datasets with dependent observations, such as those collected from the same participants across different conditions [24]. Another variant, leave-one-group-out (LOGO) CV, is also beneficial, particularly when dealing with data segmentation where observations are grouped by the participant’s identification key (ID). Moreover, overfitting, especially with small datasets, may arise during model selection from using the same validation/testing set in the hyperparameter selection and performance evaluation processes. Therefore, the nested CV approach is proposed as a mitigation strategy for selection bias and performance overestimation [25,27,50].

To address the methodological limitations identified earlier, this study adopted several best practices. Firstly, a non-overlapping segmentation approach was utilized instead of an overlapping one to minimize the impact of dependent observations. Additionally, only the most relevant features were selected within the context of stress recognition. Furthermore, the LOGO validation strategy was employed to reduce dependency and data leakage resulting from using multiple observations of the same participant. Lastly, a nested CV approach was implemented to mitigate issues related to using the same validation sets for both hyperparameter selection and performance evaluation.

## 3. Materials and Methods

### 3.1. Dataset

This study employed three datasets. The primary dataset, collected previously by the researchers, served as the training set. Two additional secondary datasets were combined and used as the testing set.

#### 3.1.1. Primary Dataset

**Figure 2:**
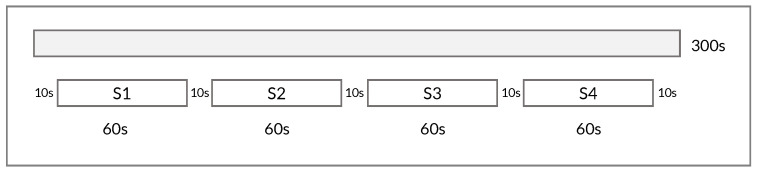
Non-overlapping segmentation of a 300 s HRV signal into 4 segments, using a window size of 60 s and a gap of 10 s between segments.

In preparation for training ML algorithms, we utilized HRV data from our prior study involving 38 participants undergoing baseline, cognitive stress, and paced breathing. Specifically, participants completed the N-back task [58], a cognitive stress test, both before and after the paced breathing exercise. The duration of HRV recordings for each condition was 5 min (300 s), obtained using a photoplethysmography (PPG)-based sensor. The experiment design, including details of the tasks and procedures, is comprehensively described in the published paper [59]. To maintain a consistent focused protocol, data from the second stress task for all participants (post-paced breathing) and the control group’s relaxed state (no paced breathing; 19 participants) were excluded. Each recording was segmented into non-overlapping 60 s windows (see Figure 2), resulting in 380 observations labeled as neutral (baseline—152), stressed (cognitive task—152), or relaxed (paced breathing—76).

#### 3.1.2. Secondary Datasets

While several publicly available datasets offered electrocardiogram (ECG) and HRV data, the selection process prioritized datasets aligning with the study’s requirements. Following a review of the datasets concerning the experiment condition, number of participants, signal length, signal quality, and study protocol, two datasets were selected for the generalizability assessment:

1. WESAD
  Wearable Stress and Affect Detection Dataset (WESAD) is a publicly available multimodal dataset consisting of physiological data recordings, including body temperature and three-axis acceleration, ECG, electrodermal activity, electromyograms, and respiration recorded during baseline, stress, meditation, and amusement conditions using chest belt and wrist sensors. Data were collected from 15 participants in a controlled laboratory experiment, and physiological signals were sampled at 700 Hz [31]. In addition, self-report surveys were administered to gauge stress and emotional states. This dataset has been widely used in relevant research studies [10,60,61,62]. All conditions except for the data collected during the amusement phase were employed in the present study.
2. SWELL
  Smart Reasoning Systems for Well-being at Home and at Work (SWELL) is a publicly available dataset collected by researchers at the Institute for Computing and Information Sciences at Radboud University [32]. It consists of computer recordings of body posture, ECG signals, facial expressions, and skin conductance from 25 participants performing two work-related tasks under two types of stress induction (i.e., receiving unexpected email interruptions and pressure to complete their work within a certain timeframe). ECG signals were sampled at 2048 Hz. In addition, the researchers collected subjective information regarding the participants’ emotions, mental effort, perceived stress, and task load. This dataset has been widely used in relevant research studies [10,63,64,65].

All HRV signals were checked for signal quality, resulting in the exclusion of one HRV recording in the relaxed state from the WESAD dataset because the number of signal samples was insufficient for HRV analysis. Moreover, the data labeled stress and relaxed for eight participants were excluded from the WESAD dataset because they performed the paced breathing exercise before the stress task. As the present study was focused on three states (i.e., neutral, stress, and relax), the HRV data collected during the amusement condition from the WESAD dataset were also excluded. Therefore, the total number of observations was 120: 38 samples were labeled neutral, 53 were labeled stress, and 29 were labeled relax.

### 3.2. Data Preprocessing

Due to the physiological differences among participants across the three datasets, all recordings were normalized based on the average HRV of each participant’s baseline measurement as shown in Equation (1) [66,67]. In this context, RR represents the HRV signal, where each RR(i) corresponds to the time interval between successive R peaks of the QRS complexes of the ECG waveform at time point *i*. Additionally, $RR{\left(i\right)}_{baseline}$ represents the HRV signal collected during the baseline phase. *N* denotes the total number of time points in the HRV signal:

(1) $$RR\left(i\right)=\frac{RR(i)}{mean(RR{\left(i\right)}_{baseline})},\;i=1,2,\;\ldots ,\;N$$

Moreover, a non-overlapping segmentation method was applied to the training dataset, dividing the 300 s HRV recording into shorter segments using a window size of 60 s and a 10 s gap to minimize dependency among segments (see Figure 2). This process yielded four segments per condition per participant. To maintain consistency between the training and testing datasets, the ECG signals from the WESAD (700 Hz) and SWELL (2048 Hz) datasets were downsampled to 500 Hz. Subsequently, peaks were detected to extract the RR intervals using the NeuroKit2 Python package [68]. Thereafter, a 300 s segment was extracted from the center of each HRV recording. The HRV signals were then normalized based on Equation (1), filtered using the adaptive threshold detection and moving average correction algorithms [69], and analyzed using the Systole Python packages [70].

### 3.3. Classification Approach

Six common supervised ML algorithms were selected: logistic regression (LR), decision trees (DT), k-nearest neighbors (KNN), Naive Bayes (NB), random forest (RF), and support vector machine (SVM). The nested CV method was used to perform hyperparameter selection and model evaluation as integral processes using the LOGO CV, which is a variation of the leave-one-out method [71]. The LOGO CV method was used to group segments resulting from the non-overlapping segmentation approach for each participant based on their ID, with each participant having data from three conditions.

**Figure A1:**
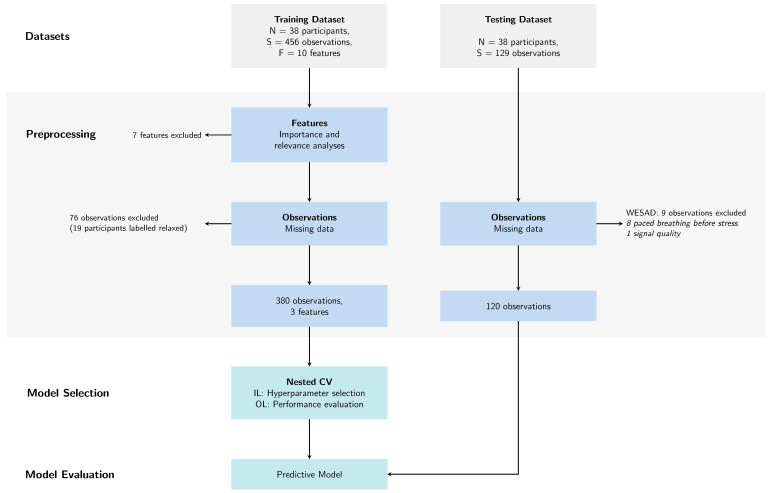
A flowchart of the ML process including dataset split, preprocessing, model selection and evaluation. Note. IL: Inner Loop, OL: Outer Loop. Adapted from [26].

For the primary dataset, the HRV data of each participant were assigned three labels based on the condition of data acquisition: (1) neutral (baseline), (2) stress (cognitive stress task), and (3) lrelax (paced breathing exercise). In a preliminary analysis of a three-class ML classifier using DT, the algorithm showed high accuracy rates in identifying the neutral (90%) and relax states (97%) but failed to distinguish the stress from neutral states (34%). This confusion between the neutral and stress states could be due to the moderate effect of the mental stressor on HRV measures as discussed in [59]. Therefore, two independent binary classifiers were implemented to differentiate the stress state from each non-stress state: (1) stress vs. neutral, and (2) stress vs. relax. To assess generalizability, the ML model that showed the best performance resulting from the nested CV method was evaluated using two combined secondary datasets (i.e., WESAD and SWELL). The ML algorithms were implemented using the Scikit-Learn Python package [72]. An illustration of the overall process, including data preprocessing, feature selection, model selection and evaluation is shown in Appendix A Figure A1.

### 3.4. Feature Selection

This study sought to distinguish between stress and non-stress states (i.e., neutral and relax). Hence, different features were selected based on the purpose of the developed ML binary classifier, albeit using a similar feature selection strategy. According to Vabalas et al. [27], the feature-to-sample ratio in limited datasets should be reasonably low. A common practice in biomedical research using small datasets is to select one feature for every 10 independent observations [24]. Thus, a maximum number of three features was selected, as the primary dataset consisted of 38 participants.

Following significant ANOVA results indicating changes in MeanRR, post hoc analysis revealed significant changes from neutral to stress (t(105) = −6.84, *p* < 0.001) and from stress to paced breathing (t(105) = 4.10, *p* < 0.001). Therefore, MeanRR was chosen as the primary feature for implementing both ML binary classifiers, as it reflected the average HRV variation and could be reliably assessed in 60 s HRV segments [73]. SDNN was selected as the secondary feature for distinguishing between stress and relaxation due to its significant statistical variation in both states, particularly in relation to paced breathing. SDNN could also be calculated from the 60 s segment [73]. To determine the significance of the remaining features, relative feature importance was calculated using an RF implemented via Scikit-Learn, which computed a weighted average score based on the degree to which the feature reduced impurity in the tree node. Based on the importance scores and their association with cardiac vagal tone [36], RMSSD and HF power were chosen for the stress vs. neutral classification. For stress vs. relax classification, SD2 was chosen due to its association with the low-frequency power and paced-breathing activities [36]. A summary of the importance scores of the selected features is outlined in Table 2. The Spearman’s rank-order correlation revealed non-significant correlation coefficients among the selected features (*p* > 0.05). As the features had different scales, a standardization approach was applied to numerical features by removing the mean value and dividing it by the standard deviation, resulting in a distribution with unit variance.

### 3.5. Nested Cross-Validation

Model selection using the CV method is divided into two main steps: hyperparameter selection and performance evaluation. These steps are often assessed using the same validation/test set, potentially leading to biased performance estimates. Nested CV addresses this by incorporating two nested CV loops. The inner loop focuses on hyperparameter selection, while the outer loop is used for the performance evaluation. A specific CV method can be selected for each loop from a pool of available methods (e.g., K-fold, leave-one-out). As previously discussed, the leave-one-out method is recommended for limited datasets and dependent observations. In this study, the LOGO method was adopted to group associated segments based on participant ID [71]. LOGO is similar to leave-one-out, but it allows for the assignment of multiple observations to a single group. The total number of splits was equal to the total number of participants in the primary dataset (38), which corresponds to a 38-K-fold CV procedure.

**Figure 3:**
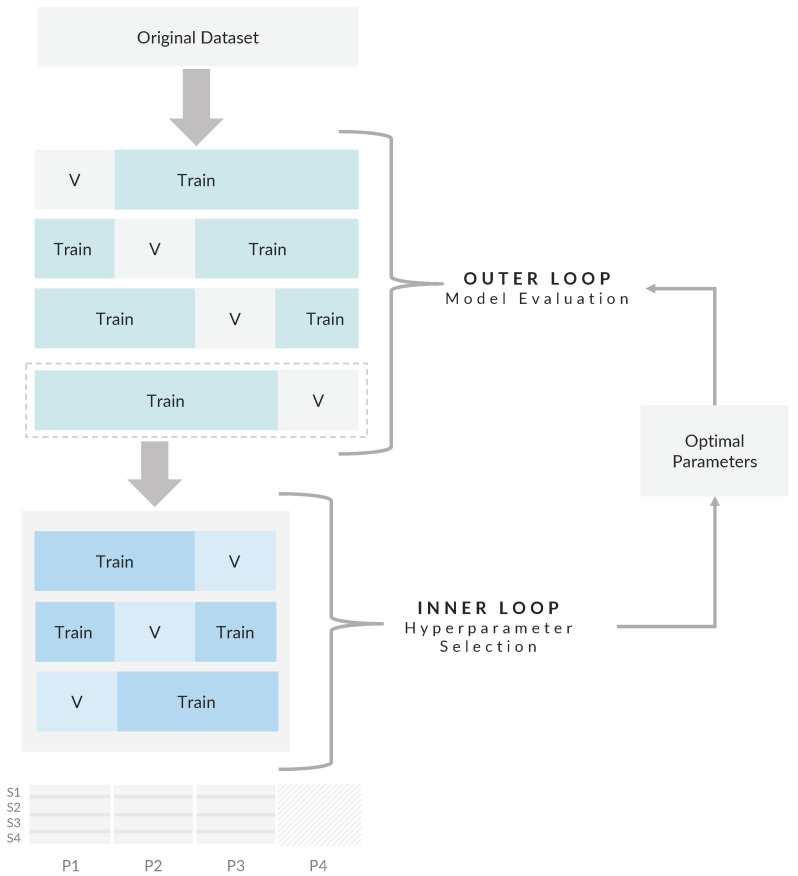
A conceptual illustration of the nested CV procedure with four participants, each with four segments. Note. V refers to the validation set, S refers to the segment number, and P refers to the participant ID.

Figure 3 illustrates the overall nested LOGO CV process using a simplified example of four participants, each with four associated segments. First, the segments are grouped based on participant ID. Then, the primary dataset is divided into N outer training/validation sets, where N is the number of participants (N = 4). Within the outer loop, a training set is selected from each iteration and passed to the inner loop for hyperparameter selection. In the inner loop, the selected training set is further divided into three (N-1) internal training/validation sets. GridSearchCV, with a predefined search space for each ML algorithm, is implemented to find the optimal hyperparameters as detailed in Appendix A Table A1. The optimal hyperparameters are then used to fit the model on the outer training set and evaluate it on the outer validation set. This process generates N performance estimates from the outer loop, from which average performance and stability metrics are calculated for each ML algorithm. Finally, the primary dataset is retrained using the model with the highest performance and stability.

While the nested CV approach aims to mitigate bias by separating the processes of hyperparameter selection and performance evaluation, the ideal scenario would involve using two entirely independent datasets. This would eliminate any potential bias or data leakage between the different stages of model selection [50,74]. However, in cases where data are limited, the nested CV approach provides a reasonable trade-off between bias mitigation and efficient use of available data.

### 3.6. Performance Metrics

ML performance was evaluated using the following metrics: accuracy, precision, recall, F1 score, confusion matrix, area under the curve (AUC), and Matthew’s correlation coefficient (MCC). Given the equal importance of correctly classifying both stressed and non-stressed states in this study, we prioritized minimizing both false positives and false negatives. Therefore, the F1-score was chosen as the primary evaluation metric. It provides a single, balanced measure by incorporating both precision and recall. Additional performance metrics were also employed for supplementary analysis, and the standard deviation (SD) was reported for the F1-score.

## 4. Results

### 4.1. Classification of Stress and Neutral States

#### 4.1.1. Model Selection

Table 3 summarizes the average performance metrics obtained using nested CV for stress vs. neutral classification on the primary dataset. Overall, the ML models had relatively low performance in classifying stress and neutral states (accuracy: 53–61%). More specifically, the precision and recall scores obtained by all models were significantly less than 70%, indicating a high misclassification rate. Among all the classifiers, RF showed the best performance and highest stability, with an F1 score of 56.2% (SD = 10.8%) and an accuracy of 61.2%. The remaining classifiers had F1 scores in the range of 43–56%. Hence, the RF with the following hyperparameters was selected for the generalizability evaluation using the secondary datasets: max_depth = 2, min_samples_leaf = 0.10.

#### 4.1.2. Generalizability Assessment

**Figure 4:**
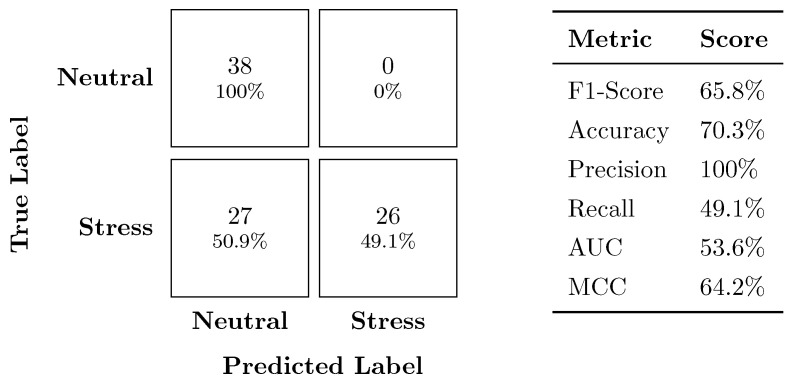
Confusion matrix and performance metrics for the stress vs. neutral classifier.

Figure 4 presents the confusion matrix with the corresponding performance metrics for the stress vs. neutral classifier on the secondary dataset. The model achieved a moderate F1-score of 65.8% and an accuracy of 70.3%. Notably, the model excelled at identifying all neutral instances (100% precision), but it had a lower recall rate for stress instances, misclassifying approximately half (49.1%).

### 4.2. Classification of Stress and Relax States

#### 4.2.1. Model Selection

Table 4 summarizes the average performance metrics obtained using nested CV for stress vs. relax classification on the primary dataset. In contrast to the stress vs. neutral classification, the models achieved relatively high accuracy rates, ranging from 84% to 89%. This suggests a better overall ability to distinguish between these states. Additionally, the precision for all models was above 80%, suggesting a lower rate of false positives compared to the classification of stress vs. neutral states. Among all classifiers, the RF demonstrated the best performance and stability, with an F1-score of 89.2% (SD = 7.2%). Notably, the RF achieved a high recall score of 96.7%, indicating good success in identifying stress instances (i.e., low false negatives). Hence, the RF was chosen for further evaluation on the secondary datasets with the following hyperparameters: max_depth = 2, min_samples_leaf = 0.10.

#### 4.2.2. Generalizability Assessment

**Figure 5:**
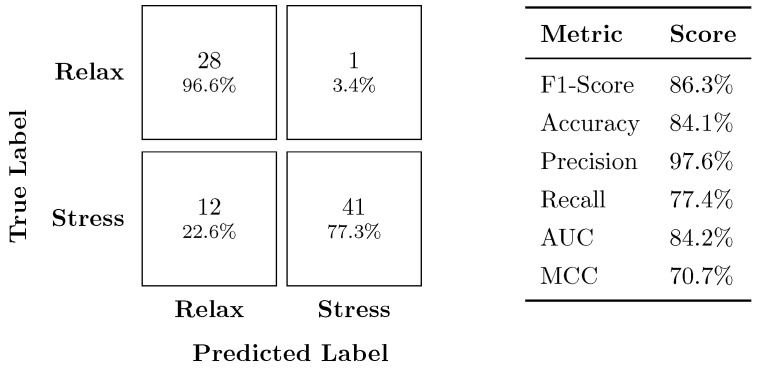
Confusion matrix and performance metrics for the stress vs. relax classifier.

Figure 5 presents the confusion matrix with the corresponding performance metrics for the stress vs. relax classifier on the secondary dataset. Compared to the stress vs. neutral classification, the model achieved significantly better performance, with an F1-score of 86.3% and accuracy of 84.1%. Notably, the model excelled at identifying relaxed instances, achieving a high precision of 97.6%. This indicates that the model rarely misclassified relaxed instances as stress. However, the recall score of 77.4% suggests that the model missed identifying some stress instances, classifying them as relaxed.

### 4.3. Effects of Validation Strategy on Model Performance

**Figure 6:**
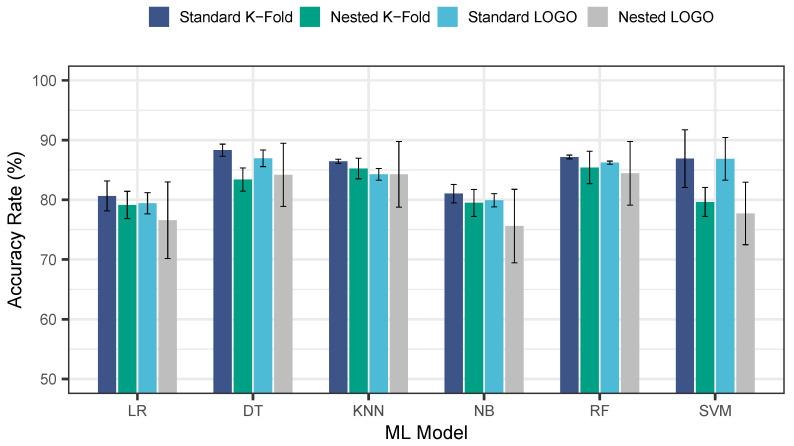
Average accuracy rate for each CV method.

To evaluate the impact of the chosen validation strategy (nested CV with LOGO) on classification performance, all ML models were compared using four different CV methods: standard K-fold CV, nested K-fold CV, standard LOGO CV, and nested LOGO CV. To ensure consistency in the K-fold CVs, all models were evaluated using 10 folds. Figure 6 illustrates the classification performance of the combined (primary and secondary) segmented dataset for the stress vs. relax classification using the accuracy metric. This analysis showcases an extreme feature selection strategy by incorporating all commonly derived HRV features from both the time and frequency domains. These features include MeanRR, RMSSD, SDNN, pNN50, LF power, HF power, LF/HF ratio, and total power.

Overall, the evaluation of different CV methods revealed that standard K-fold achieved the highest average accuracy across all investigated ML models. Nested LOGO CV, on the other hand, exhibited the lowest performance, with an average accuracy 5% lower than standard K-fold. This difference was most pronounced for the SVM model, where standard K-fold yielded a 9.2% higher accuracy compared to nested LOGO CV. The difference for the RF model was slightly smaller, around 2.8%. Furthermore, nested LOGO CV showed a higher standard deviation across all models, suggesting potential instability in its performance compared to the other CV methods.

**Figure 7:**
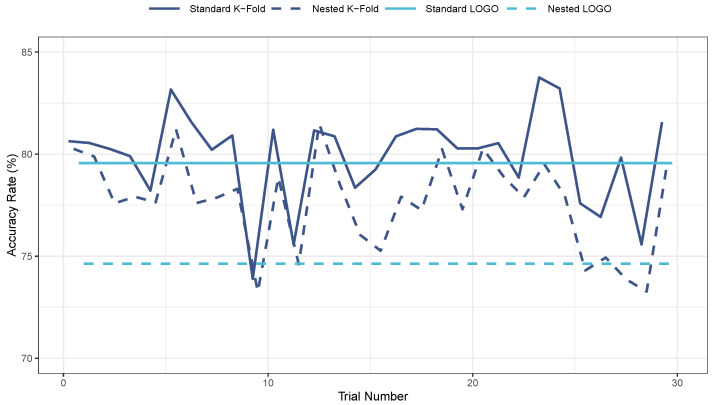
Performance of standard and nested implementations of K-fold and LOGO CV methods over 30 trials. Note. Code Adapted from Sci-kit Learn [75].

To further assess the differences in performance between the standard and nested versions of K-fold and LOGO CV methods, we conducted 30 trials focusing on the RF classifier. Each trial involved shuffling the observations and varying the seed parameter for the K-fold method. However, group randomization or shuffling was deemed unnecessary for the LOGO CV, as all observations were included in the analysis irrespective of their order. This characteristic of LOGO CV resulted in consistent performance across all trials, reflected by a flat line in Figure 7. Hyperparameter selection for the nested CV methods employed GridSearchCV within the inner loops, whereas standard CV methods utilized it in the main loops. Subsequently, the identified optimal hyperparameters were used to train the model on the training set. Notably, the standard (non-nested) implementations of both K-fold and LOGO CV generally achieved higher accuracy rates compared to their respective nested counterparts. Furthermore, the K-fold methods consistently outperformed the LOGO methods in terms of accuracy.

## 5. Discussion

The purpose of this study was to evaluate the effectiveness of supervised learning algorithms for classifying stress and relaxation levels using HRV features. We addressed limitations in existing research by developing reliable ML classifiers to mitigate overfitting, overly optimistic performance estimates, and generalizability challenges.

### 5.1. Model Performance

Two independent binary classifiers were implemented to identify stress from non-stress states (i.e., neutral and relax). Based on the nested CV model selection results, the RF achieved the highest performance among the remaining ML algorithms in terms of identifying both stress and non-stress states. In a seminal investigation of the performance of various ML classifiers, Fernández-Delgado et al. [76] assessed 179 classifiers from 17 families in 121 datasets and concluded that RF had the best performance. When deploying affect recognition in real-world settings, clinicians and users benefit from interpretable and explainable ML models [77,78]. Given that RF is based on ensemble learning of numerous decision trees, there may be a lack of understanding regarding how particular decisions were made between the predictors and the outcome [79]. Therefore, several strategies have been proposed to address this issue, including the introduction of a taxonomy of RF interpretative models via model visualization and post hoc explanatory methods [79,80]. According to the findings of the current study, DT achieved comparable performance to RF (see Table 3 and Table 4), which is considered as a simple and easy-to-understand classification algorithm in the healthcare field [81].

Generally, the RF model performed significantly better in classifying stress vs. relaxation (F1 score = 89.2%) compared to stress vs. neutral (F1 score = 56.2%). This likely reflects the stronger physiological impact of paced breathing on cardiovascular activity compared to the mild effects of mental stress tasks. Notably, the relevant HRV features used in the stress vs. relax classifier were significantly different between the two states. However, a note of caution is needed here, as the “relaxed” state in this study was associated with the paced breathing exercise itself. Future studies could benefit from measuring HRV after the breathing exercise to obtain a more accurate representation of a true relaxed state or by supplementing the data with subjective self-reported scores from participants to provide a more holistic picture of their relaxation levels [18].

### 5.2. Performance Overestimation

While our findings of the RF model performance achieved an accuracy of 60.8% in differentiating stress from neutral states, this falls short of the 80% or higher success rates reported in similar studies [16,31,82]. This performance gap may stem from two methodological factors in the reviewed studies: (1) using overlapping segmentation during data preprocessing, which can introduce dependence between observations, or (2) incorporating a high number of features relative to the dataset size, potentially leading to overfitting. Although Castaldo et al. [17] mitigated these limitations by implementing non-overlapping segmentation and utilizing a minimal feature set, they achieved a high accuracy rate of 94% with the KNN model on their primary dataset. However, a crucial consideration lies in the generalizability of their findings to a broader population due to the limited dataset size employed in their study (42 participants). In comparison, our study utilized a slightly larger dataset size (76 participants), encompassing data from both primary and secondary datasets. Generally, small training and testing sets do not represent the general population and, by extension, cannot support an accurate assessment of the generalizability of ML model performance [24].

To address potential performance overestimation during model selection, we employed the nested LOGO CV method for both hyperparameter selection and performance evaluation. Despite the variance-bias trade-off [83], this approach is only advised for small datasets, as the variance of generalization performance can be quite high otherwise. In the case of large datasets, alternative methods like leave-five-group-out CV can be employed. This approach leverages multiple groups for validation by aggregating participant-dependent observations, simulating the K-Fold method.

Overall, performance overestimation was demonstrated using a comparison of different validation strategies. Consistent with the literature [23,84,85], LOGO CV and, particularly, nested LOGO CV methods provided lower accuracy rates compared to standard and nested K-fold CV methods, with a mean difference of 5%, across the investigated ML models. Similarly, a study on human activity recognition data found that K-fold CV overestimated the accuracy of an RF classifier by 13% compared to leave-one-out CV, highlighting the importance of choosing appropriate validation strategies [86]. Performance estimates obtained through standard CV methods might exhibit susceptibility to bias, potentially leading to overestimated accuracy metrics. This issue can be attributed to two primary factors. First, standard CV methods can suffer from data leakage, as the same data are used for both hyperparameter selection and model evaluation. Second, the presence of dependent observations, either due to data segmentation or derived from the same participants, can lead to inflated performance measures [49,50,87].

### 5.3. Model Generalizability

A critical aspect of ML development is generalizability. While achieving high generalizability is desirable, establishing acceptable levels for generalization is also important [88]. Therefore, the testing phase in the present study employed two secondary datasets to evaluate how well the ML algorithms adapt to unseen data. The secondary datasets were carefully selected based on the experimental protocol and HRV recording length, but the HRV data were collected with ECG-based instruments rather than the PPG-based instruments used in the primary dataset. Additionally, participants in the SWELL dataset underwent a work-related stress task that differed slightly from the primary dataset. However, both tasks evoked a mental stress workload. Thus, the goal of the generalizability test was to assess model performance not only on unseen data but also extending the application on data collected with different instruments and under slightly different mental stressor conditions. Altogether, the RF model demonstrated good classification performance on the secondary datasets, with an F1 score of 86.3% for the stress vs. relax states. However, the model’s ability to differentiate stress from neutral states was lower, achieving an F1 score of 65.8%.

### 5.4. Limitations

Although the present study successfully demonstrated the impact of using a robust ML methodology for small datasets, it features certain limitations in terms of dependency, labeling strategy, and model stability. First, pure dependency is not necessarily implied when the violation of the independence assumption is mitigated by grouping associated segments via the LOGO CV method [89]. The observations were still interdependent within a group because they were generated from the same participant. Second, the observations were assigned to one of three classes (neutral, stress, and relax) based on the conditions under which the data were collected. In accordance with the methods employed in similar studies [41,46,52], it may have been more ecologically valid to supplement the dataset with the subjective scores reported by participants, as these reflected their current stress or relaxation levels. Lastly, the relatively high SD of the outer CV performance indicates stability issues in the LOGO CV methods. Hence, further research is needed to investigate the causes of model instability and explore approaches to better stabilize the model.

## 6. Conclusions

In conclusion, this study explored the potential of supervised learning for stress and relaxation recognition using HRV features employing binary classification models. We identified critical limitations in existing research regarding data segmentation, feature selection, and model evaluation, which can lead to overfitting and hinder generalizability. To overcome these limitations, we implemented robust ML algorithms with careful consideration of appropriate validation strategies and the selection of relevant features.

Based on our findings, the RF model achieved the best performance in distinguishing stress from non-stress states, showing notably higher accuracy in identifying stress from relaxation (F1-score: 86.3%) compared to neutral states (F1-score: 65.8%). The generalizability of this model was further demonstrated by evaluating its performance on publicly available datasets that followed a similar protocol to our primary dataset. While the performance metrics of this study may be lower than those reported in previous studies, this difference likely reflects our emphasis on implementing robust methodologies aimed at reducing the effects of overfitting and data leakage. This focus is essential not only for promoting generalizability but also for developing more interpretable and explainable ML models in the context of real-world applications, particularly when dealing with limited physiological datasets.

## Figures and Tables

**Table 1:** Heart rate variability features.

Measure	Unit	Description
Time Domain		
MeanRR	ms	Mean of all RR intervals
RMSSD	ms	Root mean square of successive differences between adjacent RR intervals
SDNN	ms	Standard deviation of RR intervals
NN50	–	Number of differences that differ by 50 ms between adjacent RR intervals
pNN50	%	Percentage of differences that differ by 50 ms between adjacent RR intervals
Frequency Domain		
ULF power	ms^2^	Power of the ultra-low frequency band: (<0.003 Hz)
VLF power	ms^2^	Power of the very low-frequency band: (0.003–0.04 Hz)
LF power	ms^2^	Power of the low-frequency band: (0.04–0.15 Hz)
HF power	ms^2^	Power of the high-frequency band: (0.15–0.4 Hz)
LF/HF	–	Ratio of LF to HF power
Total power	ms^2^	Sum of the energy in the ULF, VLF, LF, and HF bands
Non-Linear Methods		
SD1	ms	Poincaré plot standard deviation perpendicular to the line of identity
SD2	ms	Poincaré plot standard deviation along the line of identity

Note. ms: millisecond; ms^2^: milliseconds squared.

**Table 2:** Feature importance scores.

Feature	Score
**Stress vs. Neutral**	
MeanRR	37.4%
RMSSD	31.3%
HF power	31.3%
**Stress vs. Relax**	
MeanRR	31.3%
SDNN	34.8%
SD2	33.9%

**Table 3:** Nested CV performance (stress vs. neutral) (%).

Metric	F1 Score (SD)	Accuracy	Precision	Recall	AUC	MCC
LR	43.1 (17.8)	53.6	38.3	58.6	43.1	71.7
DT	54.4 (11.4)	59.5	59.9	57.9	54.4	60.0
KNN	56.3 (11.8)	57.2	58.1	61.2	56.3	62.7
NB	53.8 (14.9)	54.3	47.5	71.1	53.8	73.7
RF	56.2 (10.8)	61.2	60.1	59.9	56.2	70.7
SVM	51.2 (13.9)	56.9	57.5	56.6	51.2	65.8

**Table 4:** Nested CV performance (stress vs. relax) (%).

Metric	F1 Score (SD)	Accuracy	Precision	Recall	AUC	MCC
LR	87.2 (11.3)	85.2	89.7	90.1	87.2	76.0
DT	87.1 (10.4)	85.9	90.5	89.5	87.1	74.3
KNN	84.0 (12.5)	81.6	85.2	87.5	84.0	77.6
NB	84.4 (10.3)	79.3	80.2	94.1	84.4	83.6
RF	89.2 (7.2)	85.5	84.8	96.7	89.2	89.1
SVM	84.3 (10.4)	80.6	83.5	89.5	84.3	76.3

## Data Availability

The secondary datasets presented in this study are available publicly online.

## Appendix A. Supplementary Data

**Table A1:** Predefined hyperparameters for the GridSearchCV.

Algorithm	Hyperparameter	Value
LR	C (regularization strength)	$10^{i}$, i = [−4, 4]
DT	max_depth min_samples_leaf	[1, 2, 3, 4] [0.02, 0.04, 0.06, 0.08]
KNN	n_neighbors	[2, 3, …, 9]
NB	var_smoothing	$10^{i}$ i = [−9, 0]
RF	max_depth min_samples_leaf	[2, 3] + None [0.05, 0.10]
SVM	C (regularization strength) kernel	$10^{i}$, i = [−4, 4] Radial-basis function

## References

[1] Picard R.W., Vyzas E., Healey J. (2001). Toward machine emotional intelligence: Analysis of affective physiological state. IEEE Trans. Pattern Anal. Mach. Intell..

[2] Kroenke K., Spitzer R.L., Williams J.B. (2001). The PHQ-9: Validity of a brief depression severity measure. J. Gen. Intern. Med..

[3] Demetriou C., Ozer B.U., Essau C.A. (2015). Self-Report Questionnaires. The Encyclopedia of Clinical Psychology.

[4] Synnott J., Dietzel D., Ioannou M. (2015). A review of the polygraph: History, methodology and current status. Crime Psychol. Rev..

[5] Tutunji R., Kogias N., Kapteijns B., Krentz M., Krause F., Vassena E., Hermans E. (2023). Detecting Prolonged Stress in Real Life Using Wearable Biosensors and Ecological Momentary Assessments: Naturalistic Experimental Study. J. Med. Internet. Res..

[6] Davenport T., Kalakota R. (2019). The potential for artificial intelligence in healthcare. Future Healthc. J..

[7] Yan Y., Zhang J.W., Zang G.Y., Pu J. (2019). The primary use of artificial intelligence in cardiovascular diseases: What kind of potential role does artificial intelligence play in future medicine?. J. Geriatr. Cardiol..

[8] Kim K.H., Bang S.W., Kim S.R. (2004). Emotion recognition system using short-term monitoring of physiological signals. Med. Biol. Eng. Comput..

[9] Egger M., Ley M., Hanke S. (2019). Emotion Recognition from Physiological Signal Analysis: A Review. Electron. Notes Theor. Comput. Sci..

[10] Sarkar P., Etemad A. (2020). Self-supervised ECG Representation Learning for Emotion Recognition. IEEE Trans. Affect. Comput..

[11] Bălan O., Moise G., Moldoveanu A., Leordeanu M., Moldoveanu F. (2019). Fear level classification based on emotional dimensions and machine learning techniques. Sensors.

[12] Ihmig F.R., Antonio Gogeascoechea H., Neurohr-Parakenings F., Schäfer S.K., Lass-Hennemann J., Michael T. (2020). On-line anxiety level detection from biosignals: Machine learning based on a randomized controlled trial with spider-fearful individuals. PLoS ONE.

[13] Healey J.A., Picard R.W. (2005). Detecting stress during real-world driving tasks using physiological sensors. IEEE Trans. Intell. Transp. Syst..

[14] Zhai J., Barreto A. Stress recognition using non-invasive technology. Proceedings of the FLAIRS 2006—Proceedings of the Nineteenth International Florida Artificial Intelligence Research Society Conference.

[15] Hazer-Rau D., Zhang L., Traue H.C. (2020). A Workflow for Affective Computing and Stress Recognition from Biosignals. Eng. Proc..

[16] Bobade P., Vani M. Stress Detection with Machine Learning and Deep Learning using Multimodal Physiological Data. Proceedings of the 2nd International Conference on Inventive Research in Computing Applications, ICIRCA 2020.

[17] Castaldo R., Montesinos L., Melillo P., James C., Pecchia L. (2019). Ultra-short term HRV features as surrogates of short term HRV: A case study on mental stress detection in real life. BMC Med. Inform. Decis. Mak..

[18] Dalmeida K.M., Masala G.L. (2021). HRV Features as Viable Physiological Markers for Stress Detection Using Wearable Devices. Sensors.

[19] Theeng Tamang M.R., Sharif M.S., Al-Bayatti A.H., Alfakeeh A.S., Alsayed A.O. (2020). A machine-learning-based approach to predict the health impacts of commuting in large cities: Case study of London. Symmetry.

[20] Berntson G.G., Quigley K.S., Norman G.J., Lozano D.L., Cacioppo J.T., Tassinary L.G., Berntson G.G. (2009). Cardiovascular psychophysiology. Handbook of Psychophysiology.

[21] Malik M., Bigger J.T., Camm A.J., Kleiger R.E., Malliani A., Moss A.J., Schwartz P.J. (1996). Heart rate variability: Standards of measurement, physiological interpretation, and clinical use. Eur. Heart J..

[22] Ernst G. (2017). Heart-Rate Variability—More than Heart Beats?. Front. Public Health.

[23] Schmidt P., Reiss A., Dürichen R., Laerhoven K.V. (2019). Wearable-based affect recognition—A review. Sensors.

[24] Foster K.R., Koprowski R., Skufca J.D. (2014). Machine learning, medical diagnosis, and biomedical engineering research—Commentary. BioMed. Eng. Online.

[25] Cearns M., Hahn T., Baune B.T. (2019). Recommendations and future directions for supervised machine learning in psychiatry. Transl. Psychiatry.

[26] Stevens L.M., Mortazavi B.J., Deo R.C., Curtis L., Kao D.P. (2020). Recommendations for reporting machine learning analyses in clinical research. Circ. Cardiovasc. Qual. Outcomes.

[27] Vabalas A., Gowen E., Poliakoff E., Casson A.J. (2019). Machine learning algorithm validation with a limited sample size. PLoS ONE.

[28] Hastie T., Tibshirani R., Friedman J. (2009). The Elements of Statistical Learning.

[29] Gedam S., Paul S. (2021). A Review on Mental Stress Detection Using Wearable Sensors and Machine Learning Techniques. IEEE Access.

[30] Giannakakis G., Marias K., Tsiknakis M. A stress recognition system using HRV parameters and machine learning techniques. Proceedings of the 2019 8th International Conference on Affective Computing and Intelligent Interaction Workshops and Demos (ACIIW), IEEE.

[31] Schmidt P., Reiss A., Duerichen R., Van Laerhoven K. Introducing WeSAD, a multimodal dataset for wearable stress and affect detection. Proceedings of the ICMI 2018—Proceedings of the 2018 International Conference on Multimodal Interaction.

[32] Koldijk S., Sappelli M., Verberne S., Neerincx M.A., Kraaij W. The Swell knowledge work dataset for stress and user modeling research. Proceedings of the ICMI 2014—Proceedings of the 2014 International Conference on Multimodal Interaction.

[33] Koelstra S., Member S.S., Mü hl C., Soleymani M., Lee J.S.S., Yazdani A., Ebrahimi T., Pun T., Nijholt A., Patras I. (2012). DEAP: A database for emotion analysis; Using physiological signals. IEEE Trans. Affect. Comput..

[34] Gjoreski M., Kolenik T., Knez T., Luštrek M., Gams M., Gjoreski H., Pejović V. (2020). Datasets for cognitive load inference using wearable sensors and psychological traits. Appl. Sci..

[35] Benchekroun M., Velmovitsky P.E., Istrate D., Zalc V., Morita P.P., Lenne D. (2023). Cross Dataset Analysis for Generalizability of HRV-Based Stress Detection Models. Sensors.

[36] Shaffer F., Ginsberg J.P. (2017). An Overview of Heart Rate Variability Metrics and Norms. Front. Public Health.

[37] Casalino G., Castellano G., Kaymak U., Zaza G. Balancing Accuracy and Interpretability through Neuro-Fuzzy Models for Cardiovascular Risk Assessment. Proceedings of the 2021 IEEE Symposium Series on Computational Intelligence (SSCI).

[38] Adarsh V., Gangadharan G.R. (2024). Mental stress detection from ultra-short heart rate variability using explainable graph convolutional network with network pruning and quantisation. Mach. Learn..

[39] Vos G., Trinh K., Sarnyai Z., Rahimi Azghadi M. (2023). Generalizable machine learning for stress monitoring from wearable devices: A systematic literature review. Int. J. Med. Inform..

[40] Jadav J., Chauhan U. (2024). Heart Rate Variability Based LSTM Model for Stress Detection with Explainable AI Insights. Int. J. Intell. Syst. Appl. Eng..

[41] Chen W., Zheng S., Sun X. (2021). Introducing MDPSD, a Multimodal Dataset for Psychological Stress Detection.

[42] Oskooei A., Chau S.M., Weiss J., Sridhar A., Martínez M.R., Michel B. (2021). DeStress: Deep Learning for Unsupervised Identification of Mental Stress in Firefighters from Heart-Rate Variability (HRV) Data. Stud. Comput. Intell..

[43] Tervonen J., Pettersson K., Mäntyjärvi J. (2021). Ultra-short window length and feature importance analysis for cognitive load detection from wearable sensors. Electronics.

[44] Smets E., Casale P., Großekathöfer U., Lamichhane B., De Raedt W., Bogaerts K., Van Diest I., Van Hoof C. Comparison of Machine Learning Techniques for Psychophysiological Stress Detection. Proceedings of the Pervasive Computing Paradigms for Mental Health.

[45] Rubin J., Abreu R., Ahern S., Eldardiry H., Bobrow D.G. (2016). Time, frequency & complexity analysis for recognizing panic states from physiologic time-series. Pervasivehealth Pervasive Comput. Technol. Healthc..

[46] Petrescu L., Petrescu C., Oprea A., Mitruț O., Moise G., Moldoveanu A., Moldoveanu F. (2021). Machine Learning Methods for Fear Classification Based on Physiological Features. Sensors.

[47] Anusha A.S., Jose J., Preejith S.P., Jayaraj J., Mohanasankar S. (2018). Physiological signal based work stress detection using unobtrusive sensors. Biomed. Phys. Eng. Express.

[48] Dehghani A., Sarbishei O., Glatard T., Shihab E. (2019). A quantitative comparison of overlapping and non-overlapping sliding windows for human activity recognition using inertial sensors. Sensors.

[49] Hastie T., Tibshirani R., Friedman J. (2009). Model Assessment and Selection. The Elements of Statistical Learning.

[50] Cawley G.C., Talbot N.L. (2010). On over-fitting in model selection and subsequent selection bias in performance evaluation. J. Mach. Learn. Res..

[51] Cho D., Ham J., Oh J., Park J., Kim S., Lee N.K., Lee B. (2017). Detection of stress levels from biosignals measured in virtual reality environments using a kernel-based extreme learning machine. Sensors.

[52] Coutts L.V., Plans D., Brown A.W., Collomosse J. (2020). Deep learning with wearable based heart rate variability for prediction of mental and general health. J. Biomed. Inform..

[53] Hawkins D.M. (2004). The Problem of Overfitting. J. Chem. Inf. Comput. Sci..

[54] Ying X. (2019). An Overview of Overfitting and its Solutions. Journal of Physics: Conference Series.

[55] Castaldo R., Xu W., Melillo P., Pecchia L., Santamaria L., James C. Detection of mental stress due to oral academic examination via ultra-short-term HRV analysis. Proceedings of the Annual International Conference of the IEEE Engineering in Medicine and Biology Society, EMBS.

[56] Esterman M., Tamber-Rosenau B.J., Chiu Y.C., Yantis S. (2010). Avoiding non-independence in fMRI data analysis: Leave one subject out. NeuroImage.

[57] Remeseiro B., Bolon-Canedo V. (2019). A review of feature selection methods in medical applications. Comput. Biol. Med..

[58] Kirchner W.K. (1958). Age differences in short-term retention of rapidly changing information. J. Exp. Psychol..

[59] Bahameish M., Stockman T. (2024). Short-Term Effects of Heart Rate Variability Biofeedback on Working Memory. Appl. Psychophysiol. Biofeedback.

[60] Elzeiny S., Qaraqe M. (2020). Stress classification using photoplethysmogram-based spatial and frequency domain images. Sensors.

[61] Jiang Y., Li W., Hossain M.S., Chen M., Alelaiwi A., Al-Hammadi M. (2020). A snapshot research and implementation of multimodal information fusion for data-driven emotion recognition. Inf. Fusion.

[62] Chakraborty S., Aich S., Joo M.I., Sain M., Kim H.C. (2019). A Multichannel Convolutional Neural Network Architecture for the Detection of the State of Mind Using Physiological Signals from Wearable Devices. J. Healthc. Eng..

[63] Behinaein B., Bhatti A., Rodenburg D., Hungler P., Etemad A. (2020). A Transformer Architecture for Stress Detection from ECG.

[64] Sriramprakash S., Prasanna V.D., Murthy O.V. (2017). Stress Detection in Working People. Procedia Computer Science.

[65] Koldijk S., Neerincx M.A., Kraaij W. (2018). Detecting Work Stress in Offices by Combining Unobtrusive Sensors. IEEE Trans. Affect. Comput..

[66] Sacha J. (2013). Why should one normalize heart rate variability with respect to average heart rate. Front. Physiol..

[67] Sacha J., Pluta W. (2008). Alterations of an average heart rate change heart rate variability due to mathematical reasons. Int. J. Cardiol..

[68] Makowski D., Pham T., Lau Z.J., Brammer J.C., Lespinasse F., Pham H., Schölzel C., Chen S.H. (2021). NeuroKit2: A Python toolbox for neurophysiological signal processing. Behav. Res. Methods.

[69] Lipponen J.A., Tarvainen M.P. (2019). A robust algorithm for heart rate variability time series artefact correction using novel beat classification. J. Med. Eng. Technol..

[70] Legrand N., Allen M. (2022). Systole: A python package for cardiac signal synchrony and analysis. J. Open Source Softw..

[71] Maleki F., Muthukrishnan N., Ovens K., Reinhold C., Forghani R. (2020). Machine Learning Algorithm Validation: From Essentials to Advanced Applications and Implications for Regulatory Certification and Deployment. Neuroimaging Clin. N. Am..

[72] Barupal D.K., Fiehn O. (2019). Generating the Blood Exposome Database Using a Comprehensive Text Mining and Database Fusion Approach. Environ. Health Perspect..

[73] Shaffer F., Meehan Z.M., Zerr C.L. (2020). A Critical Review of Ultra-Short-Term Heart Rate Variability Norms Research. Front. Neurosci..

[74] Varma S., Simon R. (2006). Bias in error estimation when using cross-validation for model selection. BMC Bioinform..

[75] (2019). Nested Versus Non-Nested Cross-Validation. Scikit-Learn Developers. https://scikit-learn.org/stable/auto_examples/model_selection/plot_nested_cross_validation_iris.html.

[76] Fernández-Delgado M., Cernadas E., Barro S., Amorim D. (2014). Do we need hundreds of classifiers to solve real world classification problems?. J. Mach. Learn. Res..

[77] Adadi A., Berrada M. (2018). Peeking Inside the Black-Box: A Survey on Explainable Artificial Intelligence (XAI). IEEE Access.

[78] Du M., Liu N., Hu X. (2020). Techniques for interpretable machine learning. Commun. ACM.

[79] Aria M., Cuccurullo C., Gnasso A. (2021). A comparison among interpretative proposals for Random Forests. Mach. Learn. Appl..

[80] Haddouchi M., Berrado A. A survey of methods and tools used for interpreting Random Forest. Proceedings of the 2019 1st International Conference on Smart Systems and Data Science (ICSSD), IEEE.

[81] Podgorelec V., Kokol P., Stiglic B., Rozman I. (2002). Decision Trees: An Overview and Their Use in Medicine. J. Med. Syst..

[82] Can Y.S., Chalabianloo N., Ekiz D., Fernandez-Alvarez J., Riva G., Ersoy C. (2020). Personal Stress-Level Clustering and Decision-Level Smoothing to Enhance the Performance of Ambulatory Stress Detection with Smartwatches. IEEE Access.

[83] Hawkins D.M., Basak S.C., Mills D. (2003). Assessing model fit by cross-validation. J. Chem. Inf. Comput. Sci..

[84] Tougui I., Jilbab A., El Mhamdi J. (2021). Impact of the Choice of Cross-Validation Techniques on the Results of Machine Learning-Based Diagnostic Applications. Healthc. Inform. Res..

[85] Saeb S., Lonini L., Jayaraman A., Mohr D.C., Kording K.P. (2017). The need to approximate the use-case in clinical machine learning. GigaScience.

[86] Bragança H., Colonna J.G., Oliveira H.A.B.F., Souto E. (2022). How Validation Methodology Influences Human Activity Recognition Mobile Systems. Sensors.

[87] (2022). Nested Cross-Validation. Scikit-Learn Developers. https://inria.github.io/scikit-learn-mooc/python_scripts/cross_validation_nested.html.

[88] Futoma J., Simons M., Panch T., Doshi-Velez F., Celi L.A. (2020). The myth of generalisability in clinical research and machine learning in health care. Lancet Digit. Health.

[89] Little M.A., Varoquaux G., Saeb S., Lonini L., Jayaraman A., Mohr D.C., Kording K.P. (2017). Using and understanding cross-validation strategies. Perspectives on Saeb et al. GigaScience.

